# Neuroprotection by S-PBN in hyperglycemic ischemic brain injury in rats

**DOI:** 10.3109/03009734.2010.498592

**Published:** 2010-07-19

**Authors:** Maria Molnar, Fredrik Lennmyr

**Affiliations:** ^1^Department of Surgical Sciences, Section of Anesthesiology and Intensive Care, Uppsala University Hospital, UppsalaSweden; ^2^Department of Surgical Sciences, Section of Cardiothoracic Surgery and Anesthesiology, Uppsala University Hospital, UppsalaSweden

**Keywords:** Brain ischemia, glucose, hyperglycemia, rat, reperfusion

## Abstract

**Background:**

Hyperglycemia exacerbates focal ischemic brain damage supposedly through various mechanisms. One such mechanism is oxidative stress involving reactive oxygen and nitrogen species (RONS) production. Nitrones attenuate oxidative stress in various models of brain injury. Sodium 2-sulfophenyl-N-*tert*-butyl nitrone (S-PBN) can be administered experimentally and has been shown to be neuroprotective in experimental brain trauma.

**Aims of the study:**

We hypothesized that S-PBN might be neuroprotective in hyperglycemic focal cerebral ischemia.

**Material and methods:**

Rats were made hyperglycemic by an intraperitoneal bolus injection of glucose (2 g/kg) and then subjected to 90 min transient middle cerebral artery occlusion (MCAO). They were randomized to a therapeutic regime of S-PBN (156 mg/kg) or saline given intravenously. Neurological testing according to Bederson and tetrazolium red staining were performed after 1 day.

**Results:**

S-PBN improved the neurological performance at day 1 both in Bederson score (1.3 ± 0.8 versus 2.7 ± 0.48) and on the inclined plane (74.5% ± 4.6 (S-PBN) versus 66% ± 8.3 (control), *P* < 0.05) but did not reduce the infarct size. Physiological data did not differ between groups.

**Conclusion:**

S-PBN may improve neurological performance at short-term survival (1 day) in the present model of hyperglycemic-ischemic brain injury in rats. This effect appeared not to be primarily related to reduced infarct size.

## Introduction

Ischemic stroke is a common and life-threatening neurological disease with substantial morbidity and mortality. Coexisting hyperglycemia occurs in a majority of stroke patients (1) and further aggravates the ischemic brain injury, an observation supported by experimental (2) and clinical studies (3).

The pathogenesis for neuronal damage following ischemic insult includes multiple mechanisms ranging from anaerobic metabolism with lactic acidosis to altered cell signaling, and all have been implicated in experimental studies (4,5). Data from our studies and other laboratories demonstrate that ischemia with concomitant hyperglycemia exaggerates the activation of extracellular signal-regulated kinase (ERK) (2,5), which in turn can be activated by intermediates of reactive oxygen and nitrogen species (RONS) (6). RONS play an important role in the pathology of cerebral ischemia and therefore have been targeted with the spin-trapping agent a-phenyl-N-*tert*-butyl nitrone (PBN), whose therapeutic potential has been reported in multiple studies of ischemic brain injury (7,8). In a previous report from our laboratory it was found that PBN reduced the hyperglycemic-ischemic damage in rats (9).

Clinical interest has been directed towards sulfonated (S-) nitrones such as S-PBN and the dual-sulfonated NXY-059. S-PBN attenuates the oxidative stress in traumatic brain injury (10), and NXY-059 has shown neuroprotective properties in a primate model of permanent ischemia (11), although clinical results this far have been disappointing (12). Based on promising data on PBN in hyperglycemic-ischemic injury (9) and the compelling feasibility of sulfonated nitrones in the treatment of ischemic brain damage, we hypothesized S-PBN to be neuroprotective in focal ischemia-reperfusion with concomitant hyperglycemia in rats. To the best of our knowledge, such experiments have not been previously reported.

## Material and methods

Male Sprague-Dawley rats were subjected to left middle cerebral artery occlusion (MCAO), induced with the filament technique (13) and maintained for 90 min followed by a 1 day reperfusion. Anesthesia was induced by an intraperitoneal (IP) injection (3 mL/kg) of a 4-fold dilution of fentanyl/fluanisone (Hypnorm^®^ TM; Janssen pharmaceutica; Beerse, Belgium) and midazolam (Dormicum^®^; F. Hoffmann-La Roche AG, Basel, Switzerland, 5 mg/mL) and was maintained with doses of 0.9 mL/kg IP as needed. Prior to MCAO the rats were given an IP glucose bolus injection (2 g/kg) (14). The tail artery was catheterized for measurement of mean arterial blood pressure and blood gas sampling. A PE-50 catheter was inserted into the left femoral vein for intravenous access. Rectal temperature was kept at 37.5°C ± 0.5°C by using a heating lamp. The surgical procedure included exposure of the left carotid bifurcation, after which the small branches of the external carotid artery (ECA) were divided and the pterygopalatine artery ligated. Then, a monofilament (Ethilon 3/0; Ethicon, Cornelia, GA, USA) was inserted into the ECA and gently advanced through the internal carotid artery until a slight resistance was felt, indicating placement at the origin of the middle cerebral artery (MCA). Electroencephalogram (EEG) was recorded over the left MCA region in order to verify ischemia (9). The rats were randomized to a therapeutic regime of a sodium 2-sulfophenyl-N-*tert*-butyl nitrone (S-PBN, 156 mg/kg) (9) or saline (0.9% NaCl) given as a 60-min intravenous infusion starting after 60 min of MCAO. The randomization was kept blinded to the operator until the experiment was terminated. The Uppsala Ethical Committee of Animal Research (C69/5) approved this study.

After 1 day, muscular strength and proprioception were examined using an inclined plane (15). The maximal elevation angle was recorded as an average of three attempts prior to surgery and after 1 day. In addition, the neurological deficit was scored using a four-level scale (16), and the rats were graded as normal (= 0), forelimb flexion (= 1), decreased resistance to lateral push (= 2), or circling (= 3).

The rats were then perfused using tri-phenyl-tetrazolium-chloride red (TTC, 2%) and phosphate-buffered formaldehyde (4%, pH 7.4) in anesthesia (17). The brains were removed and sectioned into 2-mm slices. Photographs were taken from the slices for volumetric analysis, based on sections ranging 2–14 mm from the frontal brain pole. The lesions were delineated by placing free-hand regions of interest around the pale areas on TTC sections, using image analysis freeware (ImageJ 1.37 for Macintosh; NIH, Bethesda, Maryland, USA), with the investigator blinded to the identities of the sections.

### Statistical methods

Data analysis was software-assisted, using Prism version 4.03 (Graph Pad Inc., San Diego, CA). Parametric data are expressed as mean ± SD and non-parametric data as mean (range) unless otherwise stated. Differences between groups were tested for statistical significance using unpaired *t* test or Mann-Whitney *U* test for parametric and non-parametric data, respectively.

## Results

### General findings

A total of 42 rats were successfully subjected to MCAO; 23 rats were excluded due to either subarachnoid hemorrhage (SAH) on *post-mortem* examination (control = 5, S-PBN = 3) or postoperative mortality not attributed to SAH (control = 8, S-PBN = 7). Another 6 rats were excluded due to unacceptable arterial blood gases (control = 2, S-PBN = 4). The resulting 13 rats were included for analysis of infarct size and neurological tests (control = 7, S-PBN = 6). All included rats showed signs of EEG signal depression consistent with adequate filament placement approximately 3–5 min after MCAO. Physiological data are shown in Table I.

**Table I.** Physiological data: pH, arterial pO_2_, pCO_2_, base excess (BE), blood glucose (B-glucose 0 = at middle cerebral artery occlusion (MCAO); B-glucose 120 = MCAO + 120 min), blood pressure (BP), body temperature (Temp = temperature at MCAO), body weight, volume of saline substitution perioperatively. Data are shown as mean ± SD. There were no statistically significant differences between the groups.

	S-PBN	Controls
pH	7.40 ± 0.02	7.38 ± 0.03
pO_2_ (kPa)	10.3 ± 1.0	10.2 ± 0.9
pCO_2_ (kPa)	5.7 ± 0.3	6.0 ± 0.3
BE (mmol/L)	2.2 ± 1.3	2.5 ± 3.1
B-glucose 0 (mmol/L)	13.7 ± 2.9	14.7 ± 3.2
B-glucose 120 (mmol/L)	12.2 ± 1.6	11.1 ± 1.4
BP (mmHg)	105 ± 15	107 ± 7
Temp (°C)	37.5 ± 0.4	37.4 ± 0.4
Saline volume (mL)	21.3 ± 1.5	22.8 ± 1.6
Body weight (g)	279 ± 29	305 ± 69

### Neurological testing

**Figure 1.**
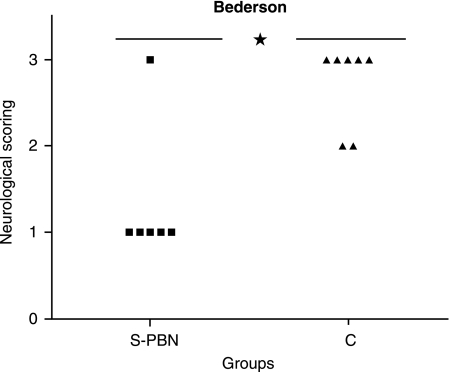
Neurological scoring according to Bederson et al. (16) (ranging from: 0 = normal, 1 = forelimb flexion, 2 = decreased resistance to lateral push, or 3 = circling) indicated a better performance in the S-PBN-treated group than in the control group. *P < 0.05 (Mann-Whitney test) (S-PBN = sodium 2-sulfophenyl-N-*tert*-butyl nitrone).

**Figure 2.**
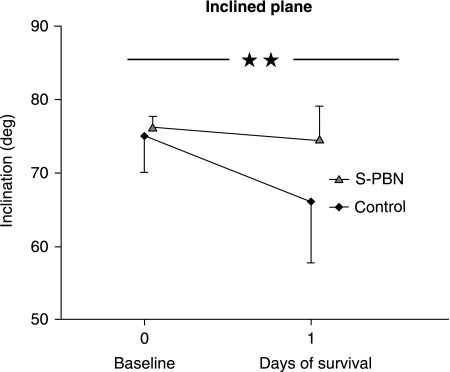
Performance on the inclined plane. The average of the three maximal angles was recorded prior to middle cerebral artery occlusion (MCAO) and after 1 day of survival. The S-PBN group managed better than the controls. Mean ± SD for 6 S-PBN-treated and 7 control rats. *P < 0.05; **P < 0.01 (unpaired *t* test) (S-PBN = sodium 2-sulfophenyl-N-*tert*-butyl nitrone).

All included rats showed contralateral motor deficit at recovery from anesthesia, indicating successful MCAO. After 1 day of survival the S-PBN-treated rats had better neurological scores than the controls (1 (1–3) versus 3 (2–3); *P* < 0.05) according to the scoring described by Bederson et al. (16) (Figure 1). The performance on the inclined plane was similar at base-line but better in the S-PBN-treated rats than controls after 1 day of survival, both in terms of absolute (74.5 ± 4.6% versus 66 ± 8.3%; *P* < 0.05) and relative changes (−1.7 ± 2.8% versus −9.0 ± 3.3%; *P* < 0.01) (Figure 2).

### Infarct size

**Figure 3.**
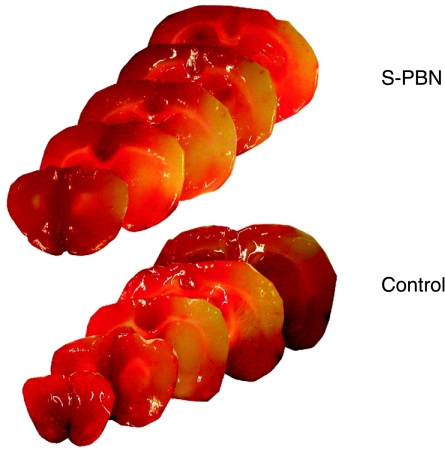
Volumetric analyses of the total brain volume and infarct size were carried out in serial 2 mm slices of brain tissue stained by tetrazolium red (TTC). Normal tissue is stained by TTC while the infarcts appear pale.

All included rats showed pallor anatomically corresponding to the MCA territory leaving normal tissue red after TTC staining (Figure 3). Integrating the areas in 2-mm slices for volumetric assessment revealed similar total brain (1054 ± 61 mm^3^ versus 1102 ± 86 mm^3^; NS) and infarct (206 ± 18 mm^3^ versus 213 ± 57 mm^3^; NS) volumes in the S-PBN-treated and the control rats.

## Discussion

The present results indicate that S-PBN may ameliorate hyperglycemic-ischemic brain damage by other mechanisms than reducing the infarct size, since only neurological performance was improved after 1 day of survival. During normoglycemia, non-sulfonated PBN reduced the infarct size by approximately 30% (18) as compared with 70% in hyperglycemic-ischemic injury (2). In our study, S-PBN improved the neurological performance but did not reduce infarct sizes, which contrasts to the more coherent data reported for PBN (8). Moreover, there was no correlation between infarct size and performance on the inclined plane (detailed data not shown). Possibly, structural differences between PBN and S-PBN might exert effects on, for instance, cerebral glucose metabolism (19) that might contribute to this discrepancy.

Under physiological conditions, glucose is the main substrate for cerebral adenosine triphospate (ATP) production and glycogen deposition (20). Normal brain function requires continuous delivery of glucose and oxygen; and the relationship between cerebral blood flow (CBF) and glucose metabolism is tightly controlled (21). The brain is to some extent capable of adapting to different glucose levels by regulation of glucose transport and metabolism; however, these mechanisms probably apply to chronic changes such as diabetes mellitus or repeated hypoglycemic events rather than acute ischemia (20).

In the present study, the induced hyperglycemia was well above the levels associated with poor stroke outcome (3,4). In line with this observation, the mortality not attributed to SAH was high among rats with successful MCAO. Interestingly, a *post-hoc* analysis of the survivors and non-survivors revealed that the only discrepant parameter between these two groups was the blood glucose level at MCAO and after 120 min. The values in the non-survivor group were 2.3 mmol/L and 3.6 mmol/L higher, respectively, at these two time point**s** (data not shown). In cerebral ischemia, hyperglycemia accelerates the ischemic brain damage, which supposedly occurs through various mechanisms such as oxidative stress, increased acidosis, mitochondrial and microvascular dysfunction, and increased inflammation (22). Recent reviews have focused on oxidative stress to explain the hyperglycemic-ischemic damage, and four main mechanisms have been identified. These are protein kinase C (PKC) activation, advanced glycation end-products (AGE), increased aldose reductase and hexosamine pathway fluxes. All of these mechanisms are associated with increased superoxide formation, which has been pointed out as the major source of oxidative stress in hyperglycemia (23).

Oxidative stress, mainly in terms of RONS, has received considerable attention in the efforts to understand the pathology of cerebral ischemia. The superoxide sources include arachidonic acid as well as xanthine oxidase metabolism and disturbed mitochondrial function (24). Furthermore, nitric oxide (NO), being a substrate for reactive compounds (24), increases in different phases of cerebral ischemia, probably as a result of the dynamic activity patterns of different neuronal and endothelial nitric oxide synthase (NOS) activities (25). Both hyperglycemia and ischemia separately result in increased loads of superoxide and NO, compounds that may be injurious per se or perhaps even more so by contributing to enhanced formation of peroxynitrite. The latter mechanism has been advocated recently in a thorough review of this field (26). It is also conceivable that hyperglycemia-induced, RONS-dependent impaired endothelial function (27) may further contribute to the ischemic process of the vasculature and the corresponding territories. Interestingly, hyperglycemia-induced depletion of endothelium and vascular muscle of nicotinamide adenine dinucleotide phosphate (NADPH) leads to reduced intracellular antioxidant capacity (28), which might in turn render the tissue vulnerable to RONS damage.

The estimated burst-like increase of RONS is approximately 4-fold upon early reperfusion after cerebral ischemia (28), and in the present study S-PBN was given primarily in order to cover this time window of anticipated RONS provocation. The neuroprotective action of the non-sulfonated PBN is incompletely known, but possibly interferes with inflammation (29) and penumbral microcirculation by promoting the recovery of metabolism (7). S-PBN is less characterized, but appears to share the spin-trapping effects of PBN *in vitro* (30). S-PBN has higher polarity and plasma clearance than PBN, and after experimental traumatic brain injury (TBI) the S-PBN penetration of the blood brain barrier (BBB) has been negated (10). Nevertheless, the beneficial effects after TBI were comparable between S-PBN and PBN regarding functional and morphological outcome (19). The explanation for this is unclear but might possibly relate to improved vascular function in the affected brain regions. Recently, S-PBN has been shown to modulate the immune cell trafficking over the BBB following TBI (31), a phenomenon in support of the notion that S-PBN affects vascular function.

The Stroke Acute Ischemic NXY-059 Study (SAINT) demonstrated that the S-PBN-related compound NXY-059 improved outcome after acute ischemic stroke (32), but disappointingly the subsequent SAINT II trial did not confirm these results (12). Methodological concerns regarding nitrone decomposition (33) might be of relevance in interpreting these discrepant findings, and in addition other shortcomings of these studies have been pointed out (34). In the case of ischemic stroke with concomitant hyperglycemia, the SAINT trials are most likely underpowered to exclude any beneficial effects of NXY-059. Thus together with the experimental support of nitrones we consider combined hyperglycemia and cerebral ischemia as an interesting target for experimental and clinical studies also in the future.
